# Supplementary material for: Sediment transport by Greenland’s icebergs
Source: Nat Commun. 2026 Jan 14;17:1172. doi: 10.1038/s41467-025-67938-4 (PMC12859035; doi:10.1038/s41467-025-67938-4)
Supplement: Supplementary file 1 — Supplementary Information [file 41467_2025_67938_MOESM1_ESM.pdf]

Supplementary Information

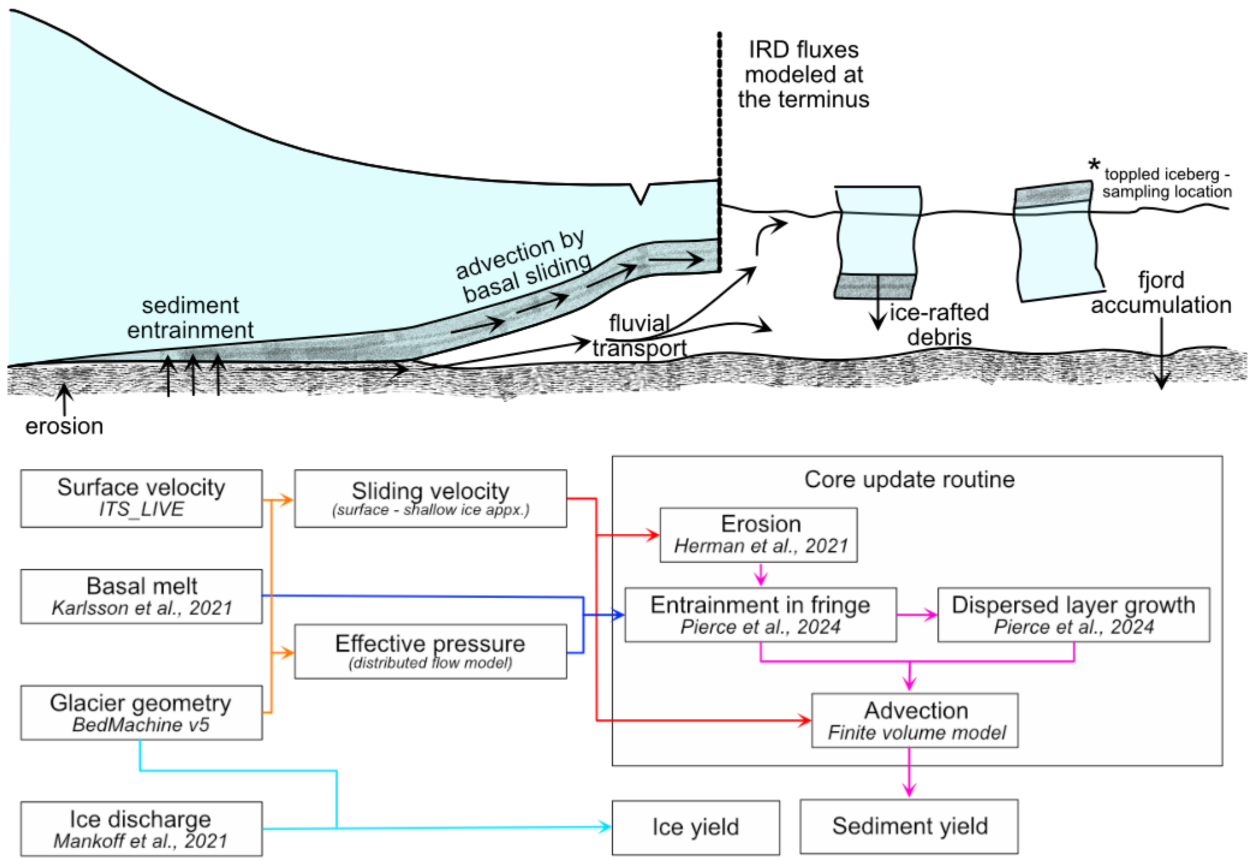

Supplementary Figure 1: Model schematic. Schematic overview of the model domain, source data, and components. Top panel shows a side view of an outlet glacier terminus, with arrows denoting sediment transport processes. IRD fluxes are measured at the terminus, shown by a dashed line. Bottom panel shows a flowchart of modeled processes and source data. The model's core update routine runs every time step, simulating erosion, vertical sediment entrainment, and lateral advection by basal sliding.

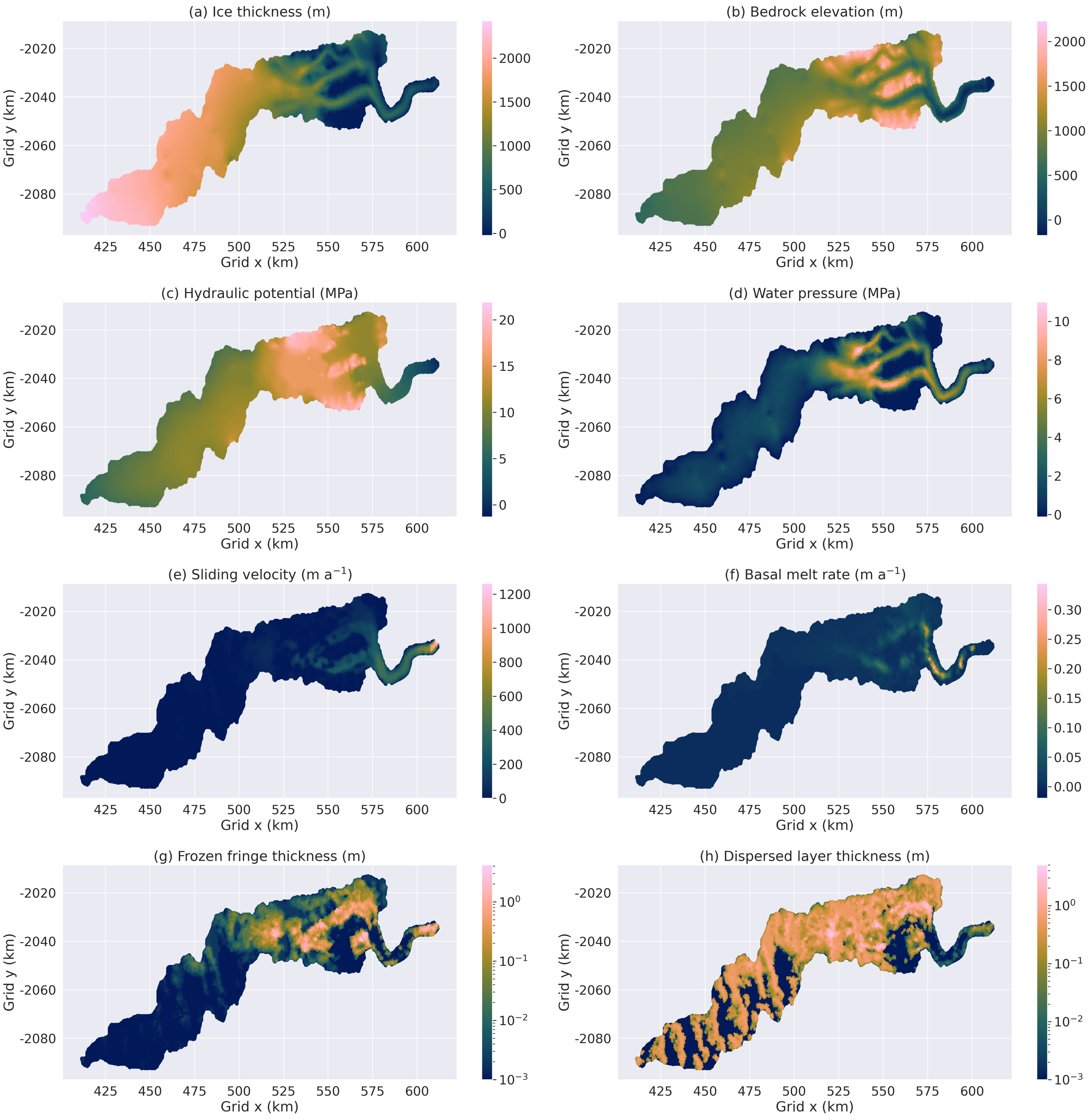

Supplementary Figure 2: Example simulation. Model fields from one example simulation over Rolige Brae, East Greenland. Grid axes are in kilometers in the NSIDC Sea Ice Polar Stereographic Projection (EPSG:3413). From top left, panels include ice thickness (a) and bedrock elevation (b) from BedMachine v5 (Morlighem et al., 2017), hydraulic potential (c) and water pressure (d) calculated as part of our distributed drainage system model, sliding velocity (e) calculated as the residual of MEaSUREs surface velocity (Gardner et al., 2022) and deformation velocity from the shallow ice approximation (see Cuffey and Paterson, 2010), and basal melt rate (f) from (Karlsson et al., 2021). The last two panels show predicted steady-state frozen fringe thickness (g) and dispersed layer thickness after 400 years of model time (h), with low values clipped to a minimum of 10^{-3} m for visualization purposes.

Parameter	Distribution	Min.	Max.	S.D.	Units
Dispersed concentration	Pareto				
Till porosity	Gaussian	0.2	0.5	0.075	
Till grain radius	Gaussian	5×10^{-5}	5×10^{-4}	5×10^{-5}	m
Fringe film thickness	Gaussian	10^{-9}	10^{-7}	5×10^{-8}	m
Critical depth	Gaussian	10	200	50	m
Ice flow coefficient	Gaussian	3.5×10^{-25}	2.4×10^{-24}	5×10^{-24}	$\mathrm{Pa}^{-n}\ \mathrm{s}^{-1}$
Hydraulic conductivity	Gaussian	0.01	0.1	0.02	$\mathrm{m\ s}^{-1}$
Erosion exponent	Gaussian	0.5	2.5	0.25	
Erosion coefficient	Derived	10^{-8}	10^{-4}		$\mathrm{m\ a}^{-1}$

Supplementary Table 1: Uncertain parameter distributions. Parameter distributions sampled during the uncertainty quantification. Erosion coefficients were derived from the randomly sampled erosion exponent, following a best-fit relationship identified for Greenland (Herman et al., 2021).
